# A New Detergent for the Effective Decellularization of Bovine and Porcine Pericardia

**DOI:** 10.3390/biomimetics7030104

**Published:** 2022-08-01

**Authors:** Martina Todesco, Saima Jalil Imran, Tiago Moderno Fortunato, Deborah Sandrin, Giulia Borile, Filippo Romanato, Martina Casarin, Germana Giuggioli, Fabio Conte, Massimo Marchesan, Gino Gerosa, Andrea Bagno

**Affiliations:** 1 Department of Industrial Engineering, University of Padua, Via Marzolo 9, 35131 Padova, Italy; martina.todesco@unipd.it; 2 L.i.f.e.L.a.b. Program, Consorzio per la Ricerca Sanitaria (CORIS), Veneto Region, Via Giustiniani 2, 35128 Padova, Italy; saima.imran@unipd.it (S.J.I.); fortunato.tiago@gmail.com (T.M.F.); sandrin.deborah@gmail.com (D.S.); giulia.borile@unipd.it (G.B.); filippo.romanato@unipd.it (F.R.); martina.casarin@unipd.it (M.C.); gino.gerosa@unipd.it (G.G.); 3 Department of Cardiac, Thoracic Vascular Sciences and Public Health, University of Padova, Via Giustiniani 2, 35128 Padova, Italy; 4 Department of Physics and Astronomy ‘G. Galilei’, University of Padova, Via Marzolo 8, 35131 Padova, Italy; 5 CNR-INFM TASC IOM National Laboratory, S.S. 14 Km 163.5, Basovizza, 34012 Trieste, Italy; 6 Department of Surgery, Oncology and Gastroenterology, University of Padova, Via Giustiniani 2, 35128 Padua, Italy; 7 Department of Prevention Veterinary Services, ULSS 3 Serenissima, P.le S.L Giustiniani 11/D, Mestre, 30174 Venice, Italy; germana.giuggioli@aulss3.veneto.it (G.G.); fabio.conte@aulss3.veneto.it (F.C.); 8 Consultant of Animal Welfare and Food Inspection, 35100 Padua, Italy; massimo_marchesan@yahoo.it

**Keywords:** tissue engineering, regenerative medicine, biomaterials, porcine pericardium, bovine pericardium, decellularization

## Abstract

Human and animal pericardia are among the most widely exploited materials suitable to repair damaged tissues in the cardiovascular surgery context. Autologous, xenogeneic (chemically treated) and homologous pericardia are largely utilized, but they do exhibit some crucial drawbacks. Any tissue treated with glutaraldehyde is known to be prone to calcification in vivo, lacks regeneration potential, has limited durability, and can result in cytotoxicity. Moreover, autologous tissues have limited availability. Decellularized biological tissues represent a promising alternative: decellularization removes cellular and nuclear components from native tissues and makes them suitable for repopulation by autologous cells upon implantation into the body. The present work aims to assess the effects of a new detergent, i.e., Tergitol, for decellularizing bovine and porcine pericardia. The decellularization procedure successfully removed cells, while preserving the histoarchitecture of the extracellular matrix. No cytotoxic effect was observed. Therefore, decellularized pericardia showed potential to be used as scaffold for cardiovascular tissue regeneration.

## 1. Introduction

In the context of regenerative medicine, there is a growing need for biomaterials that not only possess adequate biocompatibility and functional features, but are also able to integrate with and grow in the recipient’s body. To date, pericardial tissues have been used in reconstructive surgery [1,2,3] and, in particular, the main applications of bovine and porcine pericardia aim at producing bioprostheses for the surgical management of cardiovascular congenital and acquired defects [4,5,6,7,8,9,10,11,12].

When possible, the pericardium is harvested directly from the patient, but the use of autologous tissues does not represent an ideal solution: it is poorly available and it can result in tissue retraction, thickening, fibrosis and loss of pliability [13]. Synthetic materials (e.g., PTFE and PET) are an alternative to autologous tissues: indeed, they are, in a sense, inert and compatible, but do not possess the ability to regenerate and grow, leading to serious disadvantages, especially for young patients. Thrombogenicity and infectability are the major drawbacks associated with synthetic vascular substitutes [14].

To meet the need for alternative materials, the focus has turned to animals, which are a potentially unlimited source of biological tissues. At present, xenogeneic pericardial tissues, i.e., bovine and porcine, treated with glutaraldehyde (GA) are widely used in cardiothoracic and vascular surgery [15,16]. GA acts as an effective sterilant and preservative agent, conferring some advantages, such as a reduced immunogenicity and a stabilized matrix, which become more resistant to enzymatic degradation [17,18]. These advantages are counterbalanced by its cytotoxic effects, increased sensitivity to tissue calcification in vivo, and marked alterations in biomechanical features [19,20]. Therefore, GA-treated biological tissues have demonstrated limited durability, which especially affects heart valve prostheses [20,21,22,23,24,25,26].

In order to overcome all these limitations, decellularization has been suggested as a possible solution: it removes all cellular and nuclear components from the native tissues, thus reducing immunogenicity [27], while minimizing any adverse effect on the composition, biological activity, and mechanical integrity of the resulting extracellular matrix (ECM). ECM is the ultrastructure that not only determines the physical properties of each biological tissue, but also guides the behavior of resident cells modulating their functions, such as chemotaxis, proliferation, and differentiation [28,29]. Decellularized matrices have been shown to facilitate the beneficial remodeling of many tissues [30,31].

Decellularized porcine pericardium has also proved promising when combined with synthetic materials, i.e., polycarbonate urethane, improving its mechanical strength and impermeability [32,33].

The TRICOL method has been found to be effective in the decellularization of native tissues and in supporting the proliferation of human mesenchymal stem cells and endothelial cells [34,35]. This method was based on Triton X-100: in December 2012, this detergent was added to the candidate list for authorization by the European Chemical Agency (ECHA) as a substance with an equivalent level of concern due to its degradation into a byproduct with endocrine-disrupting properties [36].

The aim of this study is the evaluation of a new detergent, Tergitol 15-S-9, as a possible alternative to Triton X-100 for decellularizing bovine and porcine pericardial tissues. The decellularization procedure removed cells, while preserving the composition, structure, and mechanical features of the native ECM without any cytotoxic effect. To assess the effectiveness of this new detergent, native and decellularized samples were compared by means of physico-chemical, histomorphometric, and biomechanical analyses.

## 2. Materials and Methods

### 2.1. Pericardia Preparation, Decellularization, and Sterilization

Fresh bovine and porcine pericardia, NBPs and NPPs, respectively, of healthy animals (Holstein Friesian calves, 7 months old, weight between 300 and 350 kg; Duroc pigs, 9–14 months old and weight between 140 and 170 kg) were collected from local slaughterhouses and treated within 3 h of sacrifice. The protocols followed by the slaughterhouses were consistent with EC regulation 1099/2009 regarding animal health and protection.

Each pericardium was isolated as described by Aguiari et al. [37,38] and then decellularized following the TRICOL method published by Spina et al. [39], but using Tergitol instead of Triton X-100 with the same concentration (1–0.1% *v*/*v*) [40,41]. Tergitol was proposed by the supplier (Sigma Aldrich, Saint Louis, MO, USA) as an alternative detergent: its effectiveness was assessed by treating some biological samples (e.g., cardiovascular tissues). Preliminary results (data not shown) confirmed its capacity to remove cells and make tissues prone to the penetration of decellularizing agents.

After being decellularized, the tissues were treated with a non-specific endonuclease (Benzonase^TM^) to degrade double- and single-stranded nucleic acids [31,39]. All reagents were supplied by Sigma-Aldrich.

Decellularized bovine and porcine pericardia samples, DBPs and DPPs, respectively, were sterilized as proposed by Fidalgo et al. [35] with a two-step treatment with a cocktail of antibiotics and antimycotics (AA) and peracetic acid (PAA).

### 2.2. DNA Quantification

NBPs, NPPs, DBPs, and DPPs were cut from the whole tissues and lyophilized using SpeedVac SPD130DLX (ThermoFisher Scientific, Waltham, MA, USA). The DNA from native and decellularized samples (*n* = 9 for each group) of known masses (10–15 mg of native and decellularized bovine and porcine pericardia) was isolated, purified, and eluted using DNeasy Blood & Tissue Kit (Qiagen, Valencia, CA, USA), following the protocol provided by the manufacturer. NanoDrop and Qubit were used to quantify approximately the amount of purified DNA, which was directly measured with NanoDrop One (Thermo Scientific, Waltham, MA, USA) at 260 nm. Afterwards, it was accurately quantified with the Qubit fluorometer: 20 μL of native and decellularized tissues samples were examined using Qubit^TM^ 1X dsDNA HS Assay Kits (ThermoFisher Scientific, Waltham, MA, USA), according to the manufacturer’s instructions. Results were converted to ng DNA/mg multiplying values by dilution factor and normalized per dry tissue weight.

### 2.3. FTIR-ATR Analysis

The protein secondary structure of native and decellularized samples was studied using Fourier transform infrared spectroscopy (FTIR). NBPs, DBPs, NPPs, and DPPs flaps (10 × 10 mm^2^, *n* = 3 for each group) were cut and equilibrated for 3–4 h in deuterium oxide (Janssen, Beerse, Belgium) to reduce the contribution of interfering water bands in the amide-I region [42]. FTIR investigations were performed using the Nicolet iS-50 spectrometer (Thermo Fisher Scientific, Waltham, MA, USA) in the attenuated total reflectance (ATR) mode. The instrument was equipped with a diamond/ZnSe crystal and pressure arm. The transmittance of both samples and background was each measured using 64 scans and infrared spectra were collected within the 4000–500 cm^−1^ range, at room temperature. Spectra were then overlapped using a Matlab^®^ script (Mathworks, Natick, MA, USA) [43] to compare the composition of the investigated materials. Amide-I and amide-II bonds, respectively at 1630 cm^−1^ and 1550 cm^−1^ [40,43], were selected to evaluate the integrity of ECM proteins. Peak transmittance ratio (R) was calculated dividing the intensity of the amide-I peak by the intensity of the amide-II peak.

### 2.4. Biochemical Assay

Hydroxyproline (Hyp) and elastin were quantified in the NBPs and NPPs (control) and DBPs and DPPs samples. Proteins were extracted from lyophilized tissue samples (4–5 mg). Elastin was extracted using the Fastin™ elastin assay kit (Biocolor, Belfast, Northern Ireland), according to the manufacturer’s instructions. Collagen content was determined from the amount of Hyp that was extracted by the Hydroxyproline Assay Kit (Sigma-Aldrich, St. Louis, MO, USA), following the kit’s protocol. The absorbance of the final solution containing elastin and Hyp was measured at 513 nm and 560 nm with a Spark 10M microplate reader (Tecan, Männedorf, Switzerland). All samples were run in duplicate.

### 2.5. Histological Analysis

Pericardial morphology and integrity were assessed by hematoxylin and eosin (H&E) staining supplied by Bioptica (Milan, Italy). Staining was performed on 6 μm cryosection and native pericardium was used as the control to evaluate possible changes caused by the decellularization procedure. Samples were analyzed with an EVOS XL Core Cell Imaging System (Thermo Fisher Scientific, Waltham, MA, USA).

### 2.6. Immunofluorescence Staining

Direct and indirect immunofluorescence staining were performed on a 6 μm cryosection to evaluate the effect of decellularization on the ECM composition and to confirm the absence of nuclei. Native pericardium was used as the control. Before staining, samples were fixed in 4% (*w*/*v*) paraformaldehyde (PFA) supplied by Bioptica.

The primary antibodies used were collagen I (1:100, C2456; Sigma), elastin (1:50, ab21610, Abcam), and collagen IV (1:200, ab6586, Abcam). The controls were incubated with 1% (*w*/*v*) bovine serum albumin, instead of primary antibodies. In order to reveal the primary antibody binding, secondary antibodies were applied in separate: goat-antimouse Alexa Fluor 555 (1:300, A21422; Invitrogen) and goat anti-rabbit Alexa Fluor 555 (1:300, A27039; Invitrogen). Nuclei were stained by 4′,6-diamidino-2-phenylindole (DAPI, Invitrogen, Thermo Fisher Scientific, Waltham, MA, USA), following the producer’s instructions. Filamentous actin was fluorescently labelled with Phalloidin–Atto 647N (1:200, 65906, Sigma-Aldrich, St. Louis, MO, USA).

Images were acquired with an epifluorescence microscope Leica AF6000, connected to a Leica DC300 digital camera and equipped with LAS AF Software (Leica Micro-System, Wetzlar, Germany). Image processing was performed using the open-source ImageJ software (NIH, Bethesda, MD, USA).

### 2.7. Two-Photon Microscopy

NBPs, NPPs, DBPs, and DPPs (tissue patches) were fixed in PFA (Bioptica) for 20 min and then stored in phosphate-buffered saline (PBS, Sigma Aldrich, St. Louis, MO, USA). The samples were analyzed by two-photon microscopy in order to check the impact of the decellularization procedure on the main ECM components’ (collagen and elastin) structure by measuring the second harmonic generation (SHG) and the elastin autofluorescence signal (AutoF). SHG and elastin imaging was performed through a custom-developed multiphoton microscope, previously described by Filippi et al. [44]. Images were acquired at a fixed magnification using an Olympus 25X water immersion objective with 1.05 numerical aperture (1024 × 1024 pixels), averaged signal over 70 consecutive frames, with a pixel size of 0.43 μm. Z-stacks of different area were recorded for the native and decellularized samples of both bovine and porcine pericardia (*n* = 3). For quantitative measurements, the RAW uncompressed images were analyzed by using ImageJ software. Coherency (C) was calculated for collagen and elastin to check the local dominant fiber orientation in the Z-stack images using OrientationJ, an ImageJ plugin [45], as described by Rezakhaniha et al. [46]. The estimated parameter ranged between 0 and 1: these values indicate the absence (isotropy) and the presence (anisotropy) of a dominant orientation, respectively.

A graphic representation of the coherency level showing the organization and distribution of the fibers was provided by fast Fourier transform (FFT) analysis. The transform-based texture analysis techniques convert the image into a new form using the spatial frequency properties of the pixel intensity variations, extracting textural characteristics from the image. Highly oriented fibers in a single direction show an elliptic shape; conversely, a circular shape is due to fibers spreading in all directions [46,47].

For the 3D representation, a magnified Z-stack was acquired with the two-photon microscope by setting a pixel size of 0.108 μm. The 3D tissue structure was analyzed with a free demo version of Imaris Viewer software (Oxford Instrument) [48].

### 2.8. Biomechanical Characterization

Sample thickness was measured using a Mitutoyo digital caliber (model ID-C112XB, Mitutoyo America Co, Aurora, IL, USA) by sandwiching them between two glass slides, whose thickness was then subtracted. The biomechanical properties were assessed using a uniaxial tensile testing machine (TRAMA, IRS, Padova, Italy). All samples were cut into dog-bone-shaped specimens with a gauge length of 5 mm and 2 mm width using an in-house designed cutter. Specimens were cut along the prevailing direction of the collagen fibers during visual inspection.

Uniaxial tests were performed using two actuators and two loading cells at room temperature; samples were continuously wetted with 0.9% NaCl solution to prevent dehydration. Samples were preloaded up to 0.1 N, and then elongated (elongation rate 1 mm/s) to rupture for measuring the ultimate tensile strength (UTS) and the failure strain (FS). Engineering stress σ (MPa), strain ε (%), and tensile modulus (E) were calculated as previously described [32]. These parameters were obtained by analyzing the strain–stress curve with an in-house developed Matlab^®^ script (Mathworks, Natick, MA, USA).

### 2.9. Sterility Test

A sterility test was performed to prove the effectiveness of the sterilization treatment. This test follows the guidelines of the European Pharmacopoeia 2.6.1 for biological samples [49] that suggests a qualitative procedure to verify the absence of contamination due to bacteria and fungi. The sterility of the following samples was checked: NBPs and NPPs as positive controls, and DBPs and DPPs to determine if the decellularization process reduces tissue contamination on its own, and DBPs and DPPs after the sterilization protocol. Tissue sterilization was evaluated as previously described [35].

### 2.10. In Vitro Cytotoxicity Evaluation

The in vitro cytotoxicity of DBPs and DPPs was evaluated with a direct contact assay as described in the ISO 10993 part 5 for the evaluation of medical devices [50]. DBPs and DPPs were cut in 2 × 2 cm^2^ squared specimens and secured in homemade inserts with a culture area of 0.5 cm^2^ under in aseptic conditions. Sterilization was performed as previously described and, after PBS washings, samples (*n* = 6 for each time point) were placed in 24-well plates.

Prior to cell seeding, samples were equilibrated in fresh culture medium, DMEM (high glucose) containing 20% FBS and 1% penicillin–streptomycin, and placed in an incubator at 37 °C overnight. Human fibroblasts (BJ cell line) were expanded at 37 °C in a 5% CO_2_ incubator with a humidified atmosphere and seeded on to the samples at passage 4 at a density of 20,000 cells/cm^2^ and cultured in the scaffolds over 7 days. Samples were collected and analyzed by qualitative and quantitative assays at day 1, day 3, and day 7.

Cellular viability and metabolic activity were determined by the selective reduction in tetrazolium salt using an WST-1 assay kit (Boster Biological Technology, Pleasanton, USA). Tissue samples were incubated with WST solution 2.5% (*v*/*v*) in culture medium at 37 °C under 5% CO_2_ for 1.5 hrs. The amount of reduced WST-tetrazolium was quantified by absorption at 450 nm in the culture medium with a Spark 10M TECAN microplate reader (Tecan, Männedorf, Switzerland). DNA extraction and quantification were performed as previously described. Values were converted into ng DNA/cm^2^.

The qualitative evaluation of the investigated tissues was performed by LIVE/DEAD assay, following the kit’s protocol. Images of the samples were acquired with an Olympus IX71 fluorescence microscope (Olympus, Tokyo, Japan).

Immunofluorescence staining was performed: tissue samples were fixed with 4% (*w*/*v*) PFA and permeabilized by incubation with 0.1% *v*/*v* Triton X-100 solution for 15 min, after two PBS washes.

The samples were stained as previously described, counterstaining filamentous actin that was fluorescently labelled with Phalloidin–Atto 647N (1:200, 65906, Sigma-Aldrich, St. Louis, MO, USA) and nuclei with 4′,6-diamidino-2-phenylindole (DAPI, Invitrogen, Thermo Fisher Scientific, Waltham, MA, USA), following the producer’s instructions. Cells plated on plastic slides were used as the positive control.

## 3. Results

### 3.1. DNA Quantification

After the decellularization treatment, the amount of DNA significantly decreased as confirmed by both the Nanodrop and Qubit assays (Table 1). DBPs and DPPs showed a reduction in DNA quantity of 92.72% (*p* < 0.0001) and 97.28% (*p* < 0.0001) as measured with the Nanodrop kit. The same test was repeated with the Qubit kit, resulting in a reduction of 96.87% (*p* = 0.001) and 98.6% (*p* < 0.0001) of the DNA quantity in DBPs and DPPs, respectively.

### 3.2. FTIR Analysis

**Figure 1:**
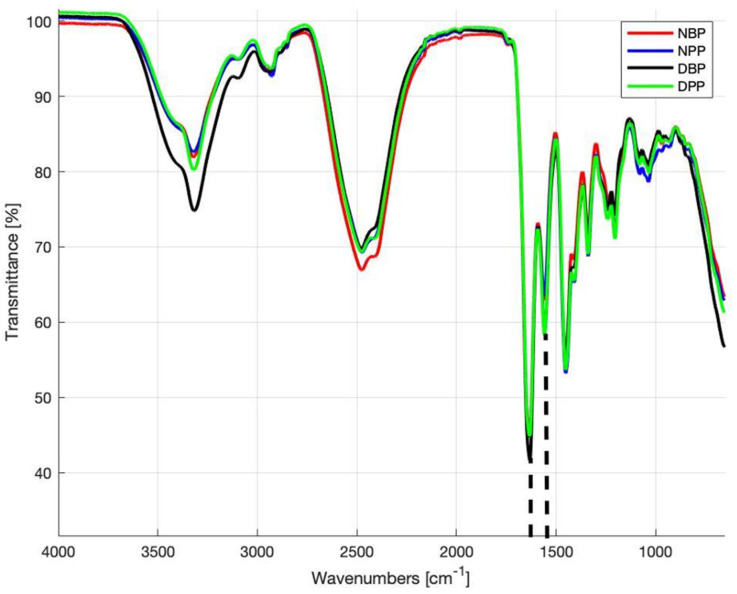
Fourier transform infrared spectroscopy attenuated total reflectance (FTIR-ATR) spectra obtained from the NBPs, NPPs, DBPs, and DPPs samples. Dashed lines identify the peaks at 1630 cm^−1^ (amide I) and 1550 cm^−1^ (amide II).

Figure 1 shows the FTIR-ATR spectra of native and decellularized bovine and porcine pericardia acquired in the frequency range of 4000–500 cm^−1^. All spectra are largely overlapped, indicating that there are no alterations in the structure of ECM proteins. The band between 3000 and 3600 cm^−1^ includes peaks of amide A due to N–H stretching (~3320 cm^−1^) and amide B caused by C–H stretching vibrations (~3020 cm^−1^) [51,52,53]. The characteristic transmittance peaks of collagen are present in the range of 1700–1250 cm^−1^: band amide I at ∼1650 cm^−1^ and amide II at ~1560 cm^−1^. The amide I band depends on the stretching vibration of the peptide carbonyl group (–CO), while amide II is due to stretching of C–N and bending of N–H. A set of three weaker bands, which represent amide III vibration modes, is centered at ∼1245 cm^−1^ [54]: it is due to N–H bending [53,54,55,56]. In the band range of 1250–1000 cm^−1^, there are typical transmittance peaks of CH and COH from carbohydrates: polycarbohydrates are the main components of glycosaminoglycans (GAGs), as reported by Jastrzebska et al. [57].

The calculated R values for NBPs, DBPs, NPPs, and DPPs were 0.72 ± 0.07, 0.71 ± 0.03, 0.71 ± 0.05, and 0.77 ± 0.07, respectively, with no statistically significant difference (*p* > 0.05) between NBPs and DBPs and between NPPs and DPPs.

### 3.3. ECM Biochemical Assessment

Elastin and Hyp contents (Table 2) in decellularized bovine and porcine pericardia were not affected by decellularization. Elastin content in the decellularized samples was lower than in the native ones, but the difference is not significant. A statistical t-test analysis was performed to compare the native and decellularized tissues: there was no significant difference (*p* > 0.05) between NBPs and DBPs and between NPPs and DPPs. DBPs apparently contain a higher amount of Hyp than the native tissue, but the difference is not significant (*p* > 0.05): this is due to the loss of cells and soluble proteins, which outweighs collagen quantity [31].

### 3.4. Histological and Immunofluorescence Analysis

**Figure 2:**
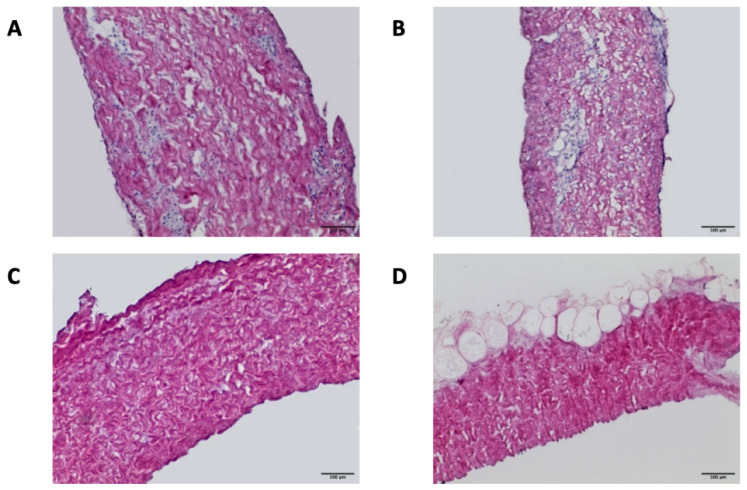
Hematoxylin and eosin staining of NBPs (**A**), DBPs (**C**), NPPs (**B**), and DPPs (**D**) samples (scale bar = 100 micron). Many nuclei are present in the native samples, while they are completely absent in the decellularized ones.

**Figure 3:**
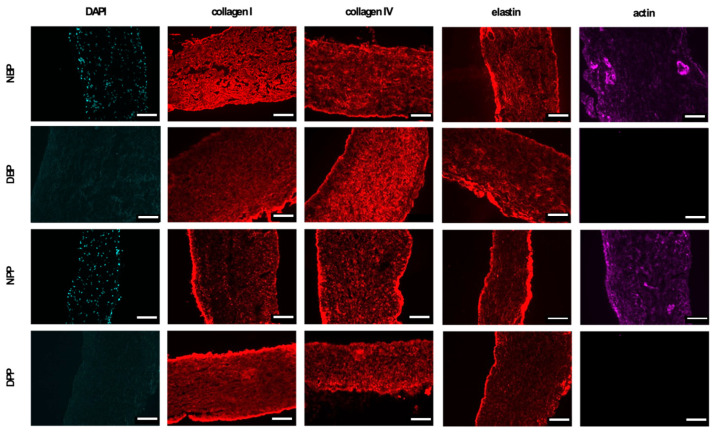
Immunofluorescence staining: rows indicate the investigated tissue and columns correspond to each specific staining (scale bar = 100 micron).

H&E staining confirmed the maintenance of the original histoarchitecture of both bovine and porcine pericardia after Tergitol decellularization and the effective removal of the cells’ nuclei. Figure 2 clearly shows that many nuclei are present in native samples, while they are completely absent in the decellularized ones. The result is confirmed by immunofluorescence staining, which shows the intact connective tissue matrix without any evidence of nuclei and actin (Figure 3). In particular, collagen I and collagen IV staining revealed that the pericardial architecture consists of multiple layers of collagen bundles with a typical wavy pattern [58]. After decellularization, no damaged fibers were visible; collagen I, collagen IV, and elastin appeared identical in the native and decellularized samples.

### 3.5. ECM Structural Assessment—Two-Photon Microscopy

**Figure 4:**
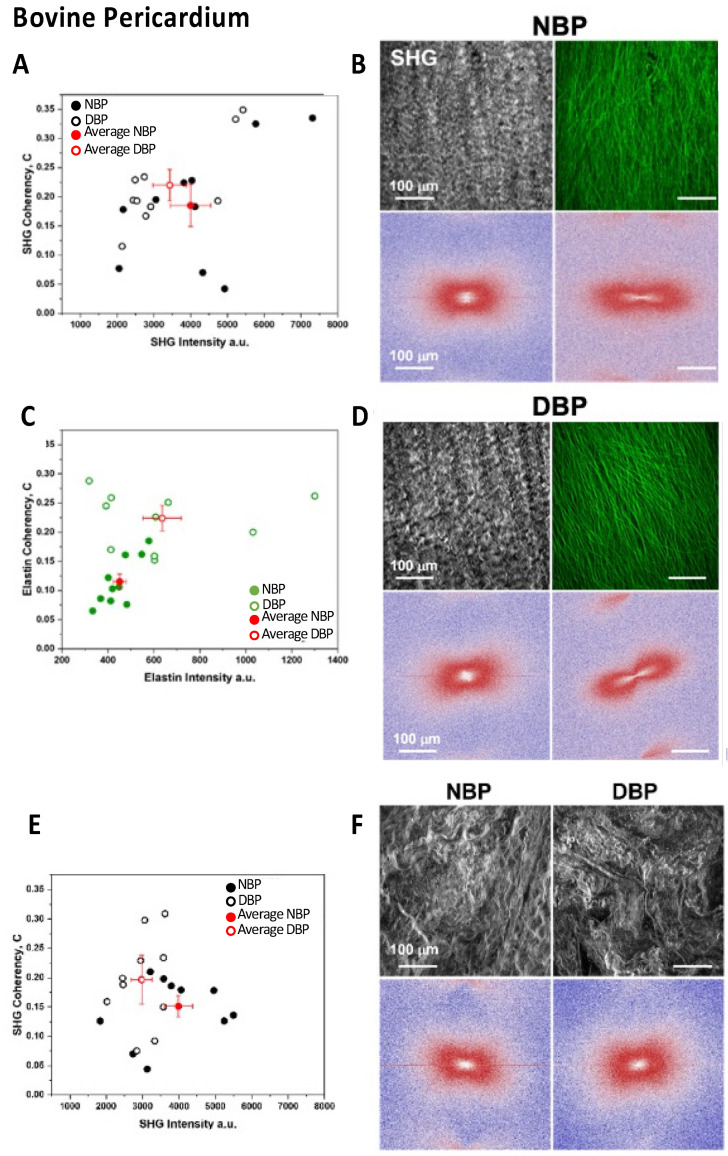

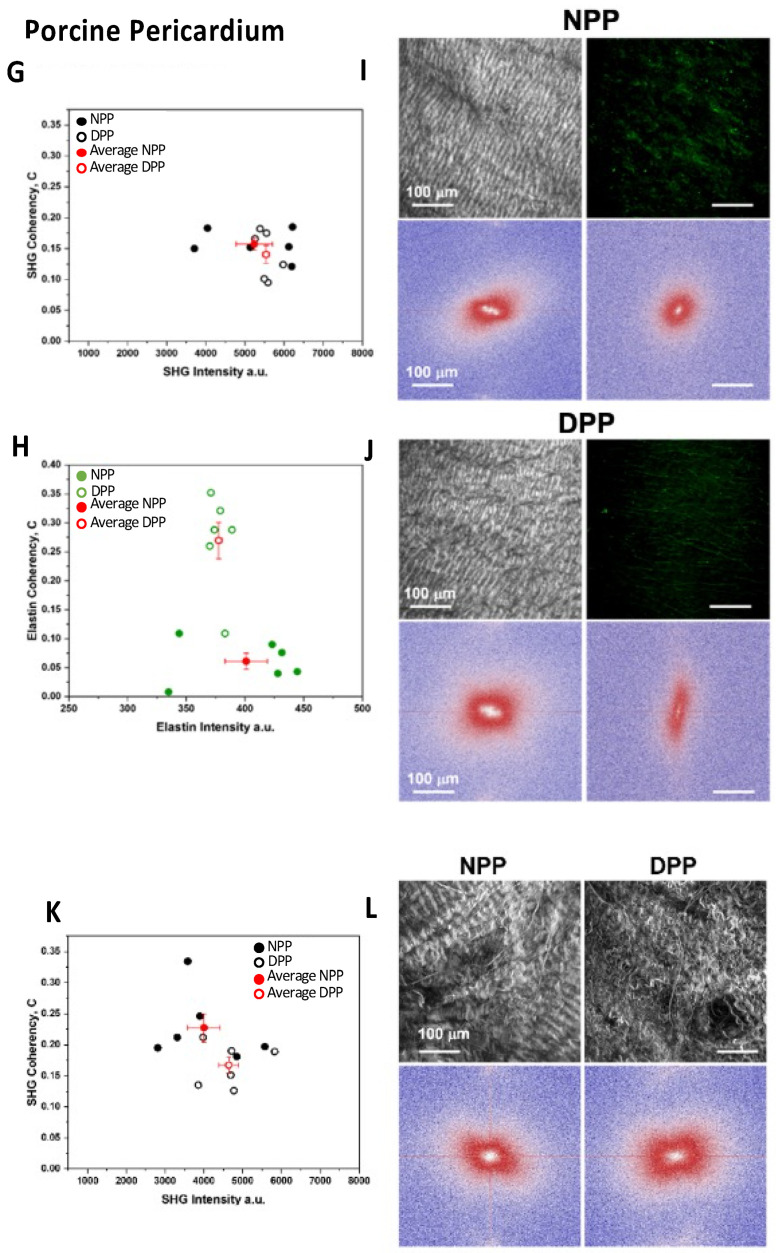
Two-photon microscopy analyses of the native and decellularized bovine and porcine pericardia. Bovine pericardium: (**A**) scatter plot of the SHG intensities vs. SHG coherency values; (**C**) scatter plot of elastin values; (**B**,**D**) images of SHG and elastin and corresponding FFT; (**E**) scatter plot of the SHG intensities vs. SHG coherency values of the serosa side; (**F**) representative images of SHG and corresponding FFT. Porcine pericardium: (**G**) scatter plot of the SHG intensities vs. SHG coherency values; (**H**) scatter plot of elastin values; (**I**,**J**) images of SHG and elastin and corresponding FFT; (**K**) scatter plot of the SHG intensities vs. SHG coherency values of the serosa side; (**L**) representative images of SHG and the corresponding FFT.

In Figure 4A,C,G,H, the scatter plots show the comparison of SHG signals vs. SHG coherency values (black points) and the corresponding elastin data (green points) of Z-stack for the different areas recorded on the NBPs, DBPs, NPPs, and DPPs samples. The average values and the corresponding standard errors (red points) were calculated. In the decellularized serosa, the SHG signal did not show a statistically significant difference comparing both NBPs and DBPs (*p* = 0.221) and NPPs and DPPs (*p* = 0.534); a similar behavior characterized the coherency values (*p* = 0.406 for NBPs and DBPs, and *p* = 0.385 for NPPs and DPPs). After decellularization, the elastin signal was maintained in both the bovine (*p* = 0.079) and porcine pericardia (*p* = 0.268); differently, coherency values were slightly higher in both bovine (*p* < 0.0001) and porcine (*p* < 0.0001) pericardia. Representative label-free images of SHG and elastin are shown with the corresponding fast Fourier transform (FFT) analysis (Figure 4B,D,I,J). FFT was similar when comparing the native and decellularized tissues, while a more ellipsoidal shape was visible for elastin in the decellularized tissues.

**Figure 5:**
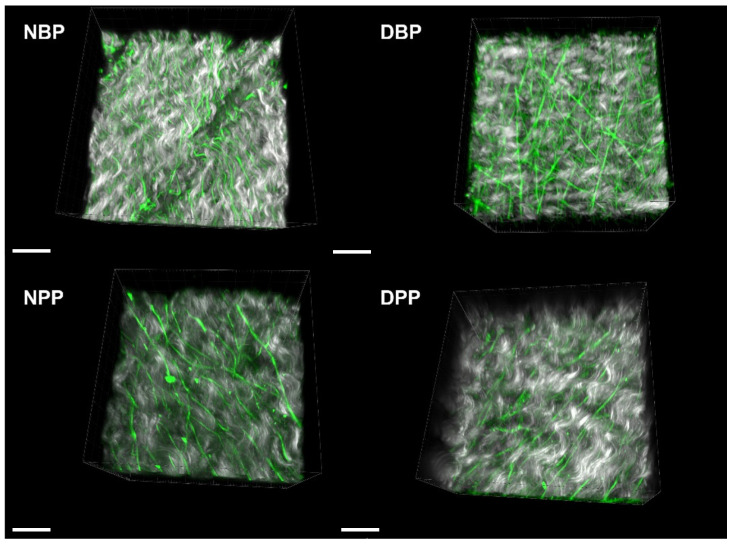
Three-dimensional representations of collagen (grey) and elastin (green) for the serosa side of NBPs, DBPs, NPPs, and DPPs. Scale bar = 20 micron.

A similar approach was applied for the fibrosa side by comparing the collagen signal and fiber direction, before and after decellularization. Figure 4E,K show the comparison of the SHG signal vs. SHG coherency values (black points) of Z-stack for the different areas recorded on NBPs/DBPs and NPPs/DPPs. The average values with the corresponding standard errors (red points) were also calculated. In the decellularized fibrosa, the SHG signal did not show statistically significant differences for both bovine (*p* = 0.061) and porcine (*p* = 0.239) tissues; a similar behavior was detectable for the coherency values (*p* = 0.130 for the native and decellularized bovine samples, and *p* = 0.0503 for the native and decellularized porcine samples). Representative label-free images of SHG are shown in Figure 4F,G,H,I,J,K,L with the corresponding FFT spectrum. FFT shapes were similar when comparing the native and decellularized tissues consistently with the coherency values. The label-free 3D representations of collagen fibers’ arrangement with elastin on the serosa side (Figure 5) demonstrate the preservation of tissue microarchitecture after Tergitol treatment for both the bovine and porcine pericardia.

### 3.6. Mechanical Characterization

Biomechanical data are summarized in Table 3. After Tergitol decellularization, the samples’ thickness decreased for the bovine and porcine pericardia, but not significantly. With regard to the maximum tension reached by pericardial samples upon loading (UTS), DBPs seemed to have a lower UTS than NBPs, whereas DPPs achieved higher UTS values than NPPs. In any case, the decellularization treatment did not result in any significant difference. FS and I values exhibited trends similar to UTS values. With regard to the Young’s modulus, E values decreased in the porcine more than in bovine tissues after decellularization, but with no significant difference.

### 3.7. Sterility Test

The sterility test was performed to check the effectiveness of the sterilization method. Antibiotic and antimycotic treatment resulted in the absence of microorganisms’ growth in DBPs and NPPs after 14 days of incubation in both media: soya-bean casein digest medium and fluid thioglycollate medium (data not shown).

### 3.8. Cytocompatibility

Once the sterility of all samples was verified, their cytocompatibility was assessed by seeding them with fibroblasts. DBPs and DPPs seeded with human fibroblasts were analyzed after 1, 3, and 7 days of incubation and tests performed, WST-1 assay and DNA extraction (quantitative assessment) and live/dead and immunofluorescence staining (qualitative assessment).

**Figure 6:**
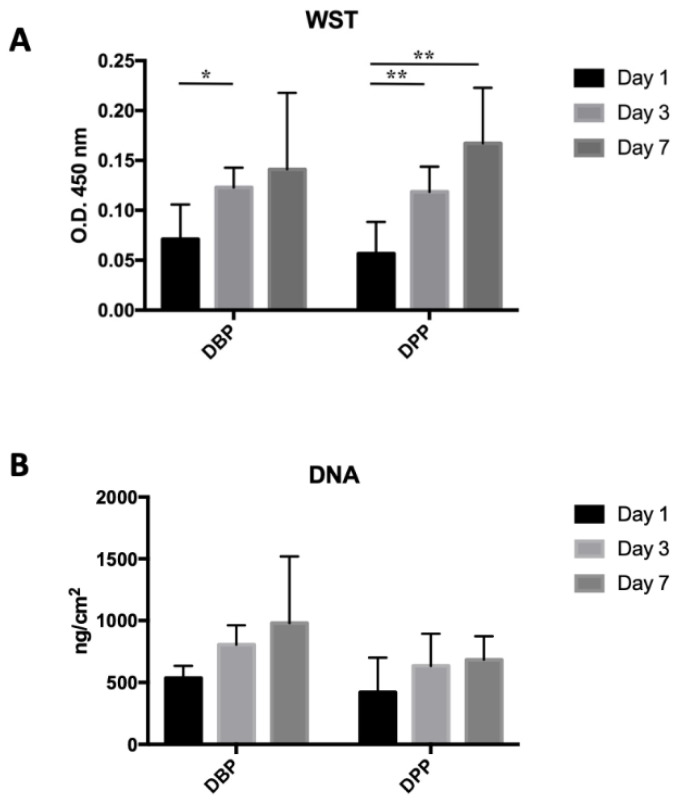
(**A**) Optical density (O.D.) values from the WST test on seeded tissues: a significant difference was present between days 1 and 3 for the bovine pericardium (* *p*< 0.05), while for the porcine pericardium, significant differences were present between days 1 and 3 and days 1 and 7 (** *p*< 0.01). (**B**) DNA extraction from decellularized tissues seeded with 20,000 cells/cm^2^: no significant difference was detected.

The WST analysis (Figure 6A) shows an increase in optical density at days 3 and 7 for both DBPs and DPPs. The increase is significant at day 3 for DBP (*p* = 0.0203), while there is no significant difference between days 1 and 7. In the case of DPPs, the increase is significant after both days 3 (*p* = 0.0092) and 7 (*p* = 0.0048) from seeding. These results were confirmed by the DNA quantification (Figure 6B): it shows an increase in the average amount of DNA/cm^2^ at days 3 (+50.3%) and 7, +50.3% and +82.59% in the bovine pericardium and +50.7% and +62.6% in the porcine pericardium, compared to day 1.

**Figure 7:**
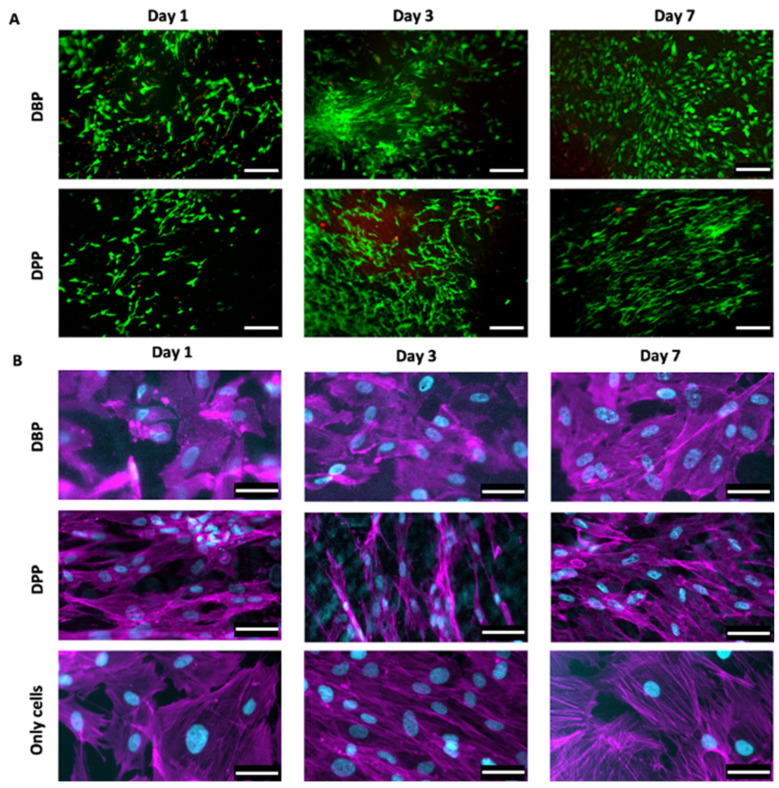
(**A**) Live/dead staining allows visualizing cells proliferation on the decellularized bovine and porcine pericardia (rows) at days 1, da 3, and 7 (columns): live cells were stained with Calcein AM (green), while dead cells were stained with ethidium homodimer-1 (red). Scale bar = 200 micron. (**B**) Immunofluorescence staining with fluorescent phalloidin (magenta) and DAPI (cyan) showed cells seeded on the decellularized bovine and porcine pericardia (rows) at days 1, 3, and 7 (columns); the third row represents cells over the control surface (plastic). Scale bar = 50 micron.

The live/dead staining (Figure 7A) highlighted the cells that were seeded over different substrates, distinguishing the live ones (in green due to Calcein staining) and the dead ones (in red due to Eth-D staining). The Eth-D signal shows a decrease over time, but it disappears at day 7; the Calcein AM signal progressively increases, indicating an expansion of live cells over both DBPs and DPPs. Both live/dead and immunofluorescence images reveal that cells tend to align following a preferential direction, particularly on the porcine pericardium; on the other hand, the cells on the bovine pericardium are more rounded, similarly to those of the control.

## 4. Discussion

Bovine and porcine pericardial patches are frequently used in cardiothoracic and vascular surgery as prosthetic materials [16,59,60,61,62]. To avoid the detrimental effects of glutaraldehyde treatment, i.e., calcific degeneration [20,39,63] and cytotoxicity [56,64], tissue engineering has been focused on tissue decellularization with the purpose of creating a material that is easy to obtain, non-immunogenic, and that can be repopulated by the patient’s own cells in order to integrate and grow with the host.

The ideal decellularization method might completely eliminate all immunogenic molecules, while not altering the native ECM from a physicochemical and structural point of view. The TRICOL method was previously used to decellularize pericardium by our research group and demonstrated effective cell removal and ECM preservation [31].

The aim of this study was the evaluation of a new detergent, Tergitol, as a possible alternative to replace Triton X-100 for decellularizing bovine and porcine pericardial tissues. The results obtained show that the decellularization protocol based on Tergitol is effective: in both the bovine and porcine decellularized tissues, nuclei are not present, and the amount of DNA is significantly reduced compared to the native counterparts, reaching values below 50 ng/mg. This is the threshold defined by Crapo et al. [29], under which the tissue should not produce an adverse immune response.

The used protocol allows us to obtain an acellular scaffold of animal origin, whose properties remain unaltered as shown by the results of the histological analysis, immunofluorescence and two-photon imaging. The main components of the ECM (collagen I, collagen IV, and elastin) were preserved after decellularization without signs of degradation or denaturation. This is also important for cell migration on ECM scaffolds, which is crucial for many biological processes, including vascular tissue endothelialization and tissue regeneration [29,65]. Human mesenchymal stem cells (hMSCs) derived from bone marrow have been reported to migrate/adhere to injured sites or implanted grafts and contribute to tissue regeneration [63,66]. It has also been shown that cells adhering to ECM can sense and respond to a wide variety of stimuli, not only chemical, but also physical, i.e., surface adhesiveness and topography, and substrate stiffness [67].

ECM proteins, such as collagen IV to which cells bind through an integrin-mediated mechanism, are mainly responsible for early and late cell migration. Cell behavior also depends on the stiffness of the material [66]: most cells respond through the organization of the cytoskeleton to the resistance they perceive with respect to the mechanical features of the substrate [67,68].

The cytotoxicity tests showed that the decellularized tissues can promote the growth of human fibroblasts and do not result in cytotoxic effects over a period of 7 days following seeding. In addition, fibroblasts seeded over the decellularized porcine pericardium, which is characterized by a greater stiffness than that of bovine origin, present a more elongated shape and are oriented along the same direction.

When comparing the effects of the Tergitol-based decellularization procedure with those previously obtained with Triton X-100 (Tricol procedure) [31], some aspects have to be mentioned. First, Triton X-100 allowed a significant reduction in the DNA content in both the bovine and porcine samples (by 97.48% and 98.43%, respectively); regarding the absolute values, Tergitol seems more effective than Triton X-100 since the final DNA amounts were lower and comparable between the bovine and porcine tissues (35.8 and 35.5 ng/mg, respectively). Moreover, while Tergitol did not cause a decrease in the samples’ thickness, Triton X-100 does, but with opposite trends for the bovine and porcine tissues: the thickness of bovine pericardium decreases, but the porcine one increases. Regarding the biomechanical features in terms of Young’s modulus, UTS, and FS, both Tergitol and Triton X-100 do not induce statistical differences with the exception of the reduction in UTS values for the decellularized porcine pericardium (*p* < 0.005). Histologically, Tergitol and Triton X-100 are both effective in removing cells, while maintaining the ultrastructural organization and biochemical composition of the extra-cellular matrix. This evidence is supported by immunofluorescence staining and microscopy investigations. In particular, the histoarchitecture of collagen and elastin is preserved; collagen bundles still exhibit their characteristic waviness after decellularization with both Tergitol and Triton X-100. In both cases, FT-IR spectra confirm the presence of ECM proteins in their native conformations after decellularization.

With regard to cytocompatibility, both detergents present promising results: an increased cell proliferation over time and the adaptation of cell shape to the tissue pattern. Fibroblasts were seeded over the Tergitol-decellularized samples: Cells were able to attach and proliferate, aligning along collagen fibers and showing a spindle-shaped morphology. This behavior was not observable in cells cultured on plastic (control) and was more evident on DPPs than DBPs. This can be due to the higher stiffness of DPPs as suggested by Rens et al. [69]. Samples decellularized with Triton X-100 were seeded with other cell types, e.g., hBM-MSCs and HUVECs: they presented spindle-shape fibroblast-like and cobblestone-like phenotypes, respectively, as well as sustained proliferation and definite polarization towards DBPs and DPPs. Since pericardial tissues are intended to be used in the cardiovascular area, further investigations will be performed with other cell types, e.g., endothelial cells.

Taken together, the results of the present study show that Tergitol can successfully replace Triton X-100 in the decellularization procedures of bovine and porcine pericardia.

Decellularized bovine and porcine pericardia are good candidates for the fabrication of biological scaffolds for tissue engineering purposes. Due to the preservation of ECM and the absence of cytotoxic effects, they can be integrated in the host organism with fundamental advantages over other materials, both allogeneic and xenogeneic, such as reduced immune reaction.

## 5. Conclusions

The present study demonstrated that the use of Tergitol detergent, instead of Triton-X 100, allows us to obtain decellularized bovine and porcine pericardia that can be used as scaffolds with regenerative potential. Tergitol-decellularized pericardial patches are non-cytotoxic and non-immunogenic, have appropriate mechanical properties, and are easily available at a low cost. These features make them good substitute materials not only in reconstructive surgeries, but also for the production of prosthetic devices, such as heart valve bioprostheses. Indeed, Tergitol decellularization allows the creation of scaffolds that are prone to be repopulated with the host’s cells: this aspect is crucial to generate many new clinical applications that require the complete integration of the prosthetic tissue with the recipient’s organism.

## Figures and Tables

**Table 1:** DNA quantification in native and decellularized bovine (NBPs and DBPs) and porcine (NPPs and DPPs) pericardia, as measured by the Nanodrop and Qubit assays. Data are expressed as mean ± SD.

	DNA Amount (ng/mg)	
	Nanodrop	Qubit
NBPs	498.1 ± 232.6	174.5 ± 125.9
DBPs	35.8 ± 13.9	5.4 ± 1.9
NPPs	1307 ± 231.2	512.4 ± 222.1
DPPs	35.5 ± 8.03	6.9 ± 3.2

**Table 2:** Elastin and hydroxyproline quantification in native and decellularized bovine (NBPs and DBPs) and porcine (NPPs and DPPs) pericardia. Data are expressed as mean ± SD.

	Elastin (μg/mg)	Hyp (μg/mg)
NBPs	117.6 ± 69.58	100.5 ± 43.82
DBPs	104.8 ± 17.84	121.9 ± 50.85
NPPs	190.6 ± 46.39	116.6 ± 40.31
DPPs	166 ± 42.49	113.9 ± 49.5

**Table 3:** Biomechanical properties of the native and decellularized biological tissues ^1^.

	Thickness (mm)	E (MPa)	FS (%)	UTS (MPa)	I (MPa)
NBPs	0.29 ± 0.04	8.54 ± 3.33	108.61 ± 20.79	31.64 ± 6.93	17.08 ± 6.22
DBPs	0.28 ± 0.06	8.72 ± 4.49	103.056 ± 19.82	26.11 ± 8.76	13.85 ± 5.58
NPPs	0.14 ± 0.03	15.23 ± 9.06	82.34 ± 37.65	15.61 ± 6.1	7.66 ± 4.35
DPPs	0.13 ± 0.02	12.71 ± 3.51	98.29 ± 20.52	19.21 ± 5.17	10.23 ± 4.35

^1^ Thickness (mm), Young’s modulus (E, MPa), failure strain (FS, %), ultimate tensile strength (UTS, MPa), and toughness (I, MPa) are reported. Data are expressed as mean ± SD.

## Data Availability

Data are contained within the article.
